# Sequential tirofiban infusions combined with endovascular treatment may improve outcomes in acute ischemic stroke - a meta-analysis

**DOI:** 10.18632/aging.202473

**Published:** 2021-02-11

**Authors:** Hongchen Zhao, Yiwei Feng, Xiaoming Rong, Yiting Mao, Zigao Wang, Yifeng Ling, Qiang Dong, Wenjie Cao

**Affiliations:** 1 Department of Neurology, Huashan Hospital, Fudan University, Shanghai, China; 2 Department of Neurology, Sun Yat-sen Memorial Hospital, Sun Yat-sen University, Guangzhou, China; 3 State Key Laboratory of Medical Neurobiology, Fudan University, Shanghai, China

**Keywords:** tirofiban, endovascular treatment, acute ischemic stroke, infarction, meta-analysis

## Abstract

In this meta-analysis, we explored whether tirofiban could safely improve outcomes when combined with endovascular therapy in acute ischemic stroke with large vessel occlusion. We searched the PubMed, EMBASE, Web of Science, and The Cochrane Library databases from January 2000 to October 2019 for relevant RCTs/non-RCTs. A total of 13 trials involving 2584 patients, of whom 893 (34.5%) received tirofiban, were ultimately included in the meta-analysis. The results suggested that tirofiban improved patient independence at 90 days (51.2% vs 42.4%; OR 1.26; *p* =0.02) without increasing the risk of symptomatic intracranial hemorrhage (OR 1.01; *p* =0.96) or mortality (OR 0.86; *p* =0.09). There was no association between the use of tirofiban and recanalization rate (OR 1.35; *p* =0.11). Subgroup analysis showed that a loading dose followed by maintenance doses, but not a single dose, of tirofiban increased favorable 90-day functional outcomes (OR 1.49; *p* =0.0008). Moreover, low maintenance doses may be more effective than high maintenance doses (OR 1.41; *p* =0.02). These results suggest that adjunctive tirofiban treatment administered as a loading dose followed by low-dose maintenance may improve functional outcomes of endovascular therapy in acute ischemic stroke.

## INTRODUCTION

Tirofiban, a glycoprotein IIb/IIIa receptor inhibitor, reduces fibrinogen-dependent platelet aggregation and subsequent thrombosis. Furthermore, glycoprotein IIb/IIIa antagonists are beneficial for high-risk non-ST elevation acute coronary syndrome patients when percutaneous coronary intervention is planned [1, 2].

Although growing evidence demonstrate that mechanical thrombectomy may improve neurological functional outcomes for ischemic stroke patients with large vessel occlusion [3–11], one third to half of patients still fail to return to an independent lifestyle due in part to refractory occlusion, re-occlusion, and distal embolism. Adjunctive therapies, including angioplasty, intra-artery thrombolysis, and GP IIb/IIIa antagonists can improve recanalization rates in culprit occlusive vessels. However, conflicting results have been obtained regarding the efficacy and safety of tirofiban in acute ischemic stroke after endovascular treatment (EVT) [12–15].

In this study, we therefore performed a thorough systematic review of previous studies comparing tirofiban vs. standard medical treatments in acute ischemic stroke patients with large vessel occlusion undergoing EVT.

## RESULTS

### Search results and study selection

**Figure 1:**
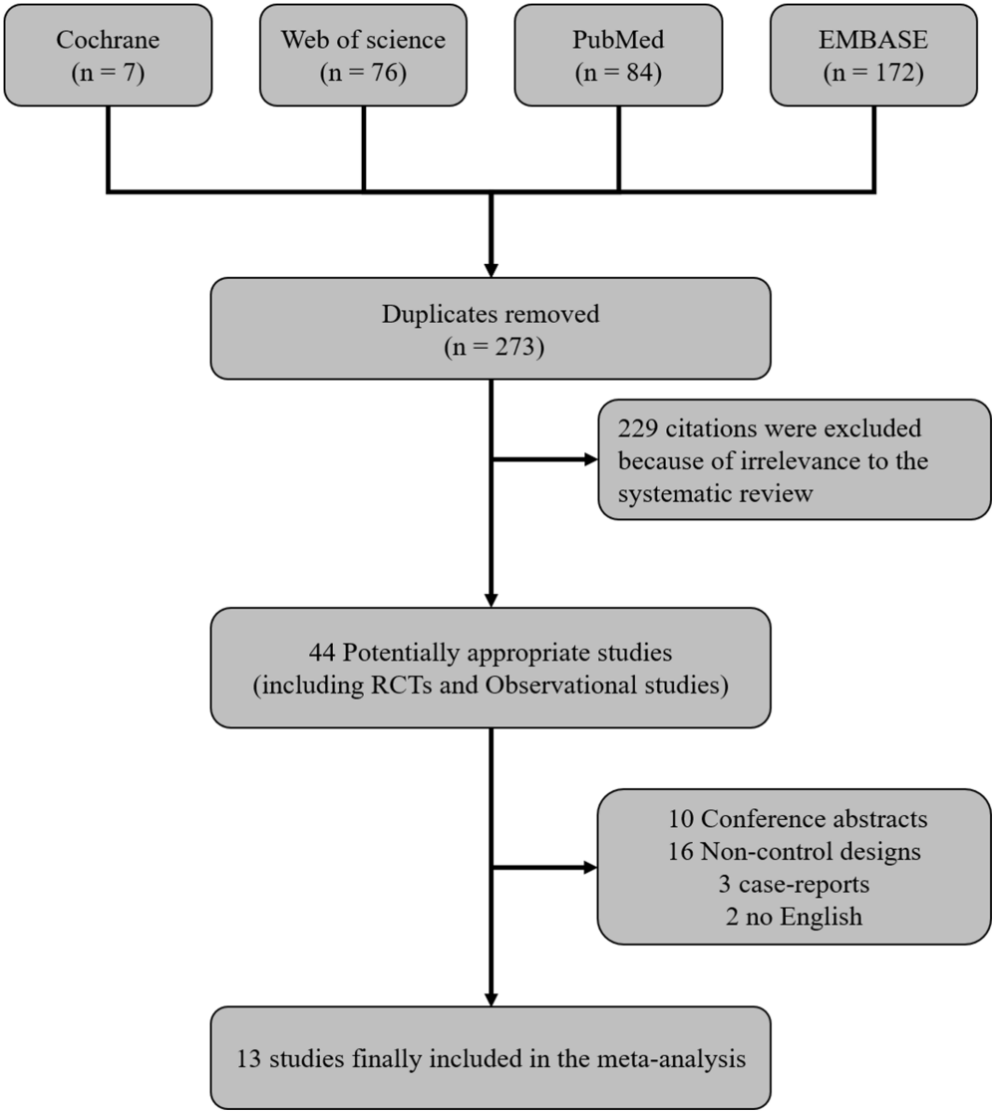
**Flow diagram showing the systematic literature search strategy and indicating the inclusion and exclusion criteria.**

Database searches identified 339 citations; 66 duplicate references were then removed. Examination of the titles and abstracts of the shortlisted citations resulted in the exclusion of an additional 229 papers. The 44 remaining studies were comprehensively examined in their entirety for compliance with the inclusion/exclusion criteria. Ultimately, 13 eligible studies were included in this meta-analysis (Figure 1) [12–14, 16–25].

### Study characteristics

A total of 2584 patients from 13 studies (1 RCT and 12 non-RCTs) were included in the final analysis; 893 (34.5%) of these patients were treated with tirofiban.

The following data were collected from each of the included studies: study design, location, time period, endovascular device used, intravenous thrombolysis, tirofiban application, time from symptom onset to groin puncture/recanalization, recanalization outcome, age, and baseline NIHSS (Supplementary Table 1). The majority of culprit vessels were located in the anterior circulation (n =2110, 81.7%). Intravenous thrombolysis bridging treatment with EVT was addressed in 9 of 13 studies. With two exceptions, rates of intravenous thrombolysis did not differ significantly between patients treated with or without tirofiban in these studies; the exceptions were the studies by Pan X et al. and Wu YF et al. [12, 19]. In those two, fewer tirofiban patients had received intravenous thrombolysis than in the other studies. Mechanical thrombectomy or direct contact aspiration was performed as first-line EVT in 12 studies, and emergent carotid artery stenting was performed within 6 h of symptom onset or in the case of penumbra reservation indicated by Computed Tomography Perfusion (CTP) in 1 study [21]. Of the 12 non-RCTs, 9 addressed indications of tirofiban, which included angioplasty, residual moderate stenosis to occlusion in culprit vessels, and intimal membrane injury [17–19, 21–23]. The other 3 studies did not address the exact indications of tirofiban [12–14, 20]. The studies were divided into the following two groups based on tirofiban dosage: 1) single dose administration (4 studies) [12, 17, 20, 22]; 2) standard administration (loading + maintenance): loading dose followed by low-dose maintenance (5 studies) [13, 18, 21, 23, 25] or high-dose maintenance (2 studies) [16, 19]. Dosage details were not available in the studies by Kellert et al. [14] and Sun C et al. [26].

### Primary endpoint

**Figure 2:**
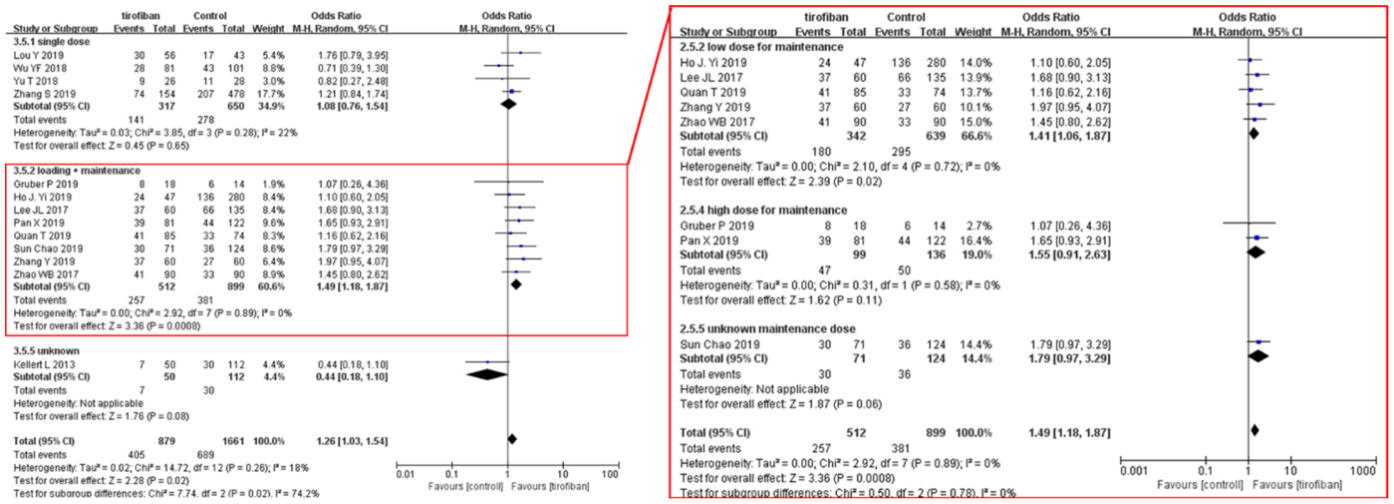
**Forest plot comparing 90-day favorable functional outcomes for EVT+ tirofiban vs. EVT.** Included trials are divided into subgroups based on tirofiban administration strategy (single dose or loading dose plus maintenance doses). Subsequent subgroup analysis was completed by separating loading dose plus maintenance studies based on the maintenance dosage (low dose, high dose, or unknown). CI, confidence interval; Weight, statistical weight (an indirect estimate of study precision and impact on overall pooled estimates of the single study result).

Favorable functional outcomes at 90 days were significantly more likely in groups treated with tirofiban (51.2% vs 42.4%; OR, 1.26 [95% CI, 1.03, 1.54]; *p* =0.02). No significant heterogeneity was detected among the trials included in this analysis (χ^2^ =14.72; *p* =0.26, *I^2^* =18%). However, there was a significant difference between the treatment subgroups (χ^2^ =7.74; *p* =0.02; *I*^2^ =74.2%) (Figure 2). Subgroup analysis showed that standard tirofiban administration (a loading dose followed by maintenance doses) improved functional outcomes compared to a single dose (OR 1.49; 95% CI 1.18–1.89; *p* =0.0008). Moreover, low-dose maintenance specifically was associated with a significant increase in favorable functional outcomes (OR, 1.41 [95% CI, 1.06 - 1.87], *p* =0.02); there was also a trend towards increased patient independence in the high-dose maintenance group that did not reach statistical significance (OR, 1.55 [95% CI, 0.91 - 2.63], *p* =0.11).

### Secondary efficacy outcome

**Figure 3:**
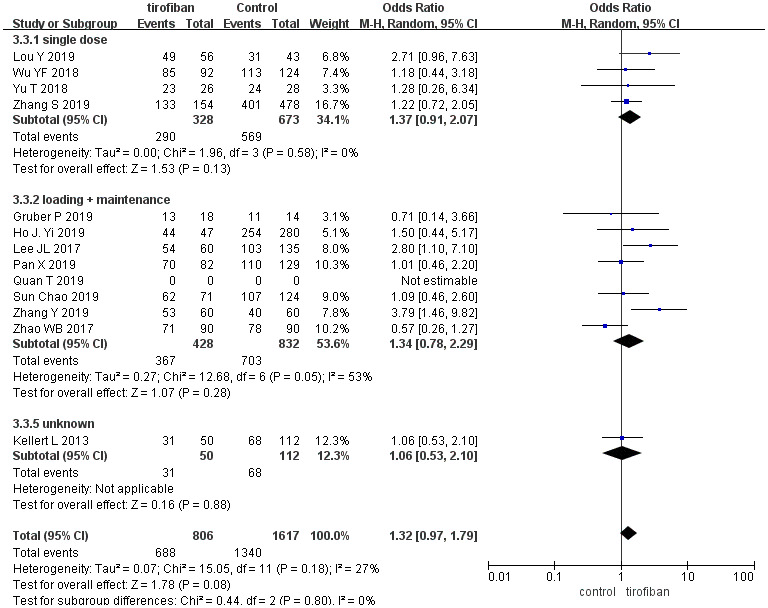
**Forest plot comparing recanalization rates for EVT+ tirofiban vs. EVT.** Included trials are divided into subgroups based on tirofiban administration strategy (single dose or loading dose plus maintenance doses). CI, confidence interval; Weight, statistical weight (an indirect estimate of study precision and impact on overall pooled estimates of the single study result).

Recanalization rates were similar between patients treated with or without tirofiban both in the overall pooled meta-analysis and in subgroup analysis (OR =1.32, [95%CI, 0.97 - 1.79], *p =*0.11). No significant differences were identified between subgroups (χ^2^=7.74; *p* =0.10, *I^2^* =39%) (Figure 3).

### Safety outcomes

### Mortality

**Figure 4:**
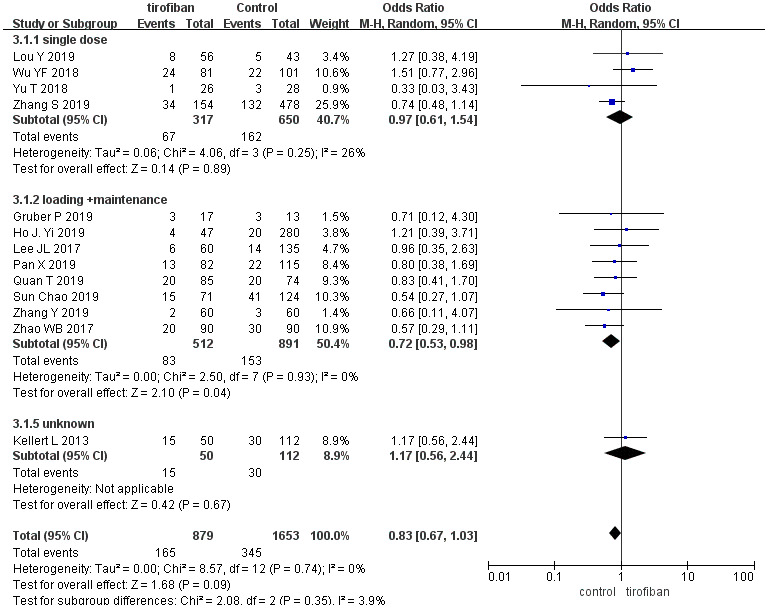
**Forest plot comparing 90-day mortality for EVT+ tirofiban vs. EVT.**

Overall pooled effect estimate analysis revealed no significant differences in 90-day mortality between patients treated with or without tirofiban (OR =0.83, [95%CI, 0.67 - 1.03], *p =*0.09), which was consistent with the subgroup analysis. No differences were found between the subgroups (χ^2^ =2.08; *p* =0.35, *I^2^* =3.9%) (Figure 4).

### Symptomatic intracranial hemorrhage (sICH)

**Figure 5:**
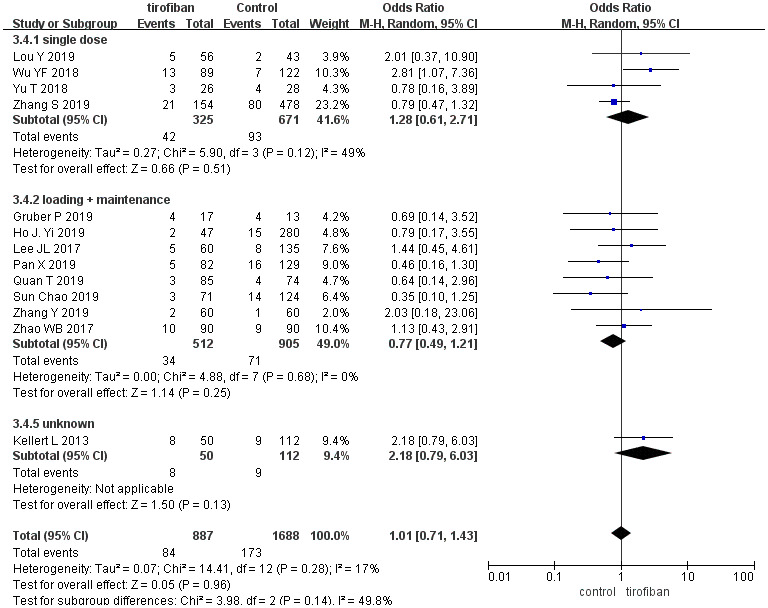
**Forest plot comparing sICH for EVT+ tirofiban vs. EVT.**

The use of tirofiban did not increase the rate of sICH (9.5% vs. 10.2%, OR =1.01, [95%CI, 0.71 - 1.43], *p* =0.96), and no differences in sICH were found between the subgroups (χ2 =3.98; *p* =0.14, *I^2^* =49.8%] (Figure 5).

### Sensitivity analysis and publication bias

Sensitivity analysis results were consistent with the pooled analysis results both for efficacy outcome as indicated by favorable functional outcomes (Supplementary Figure 1) and recanalization rates (Supplementary Figure 2) as well as for safety outcomes as indicated by sICH rates (Supplementary Figure 3); however, after removing the study reported by Wu et al [12] or Kellert et al [14], patients treated with tirofiban and EVT had a lower rate of 90-day mortality than those treated with EVT alone (Supplementary Figure 4). In funnel plot analysis, the shape of the plot did not indicate obvious asymmetry upon visual inspection (Supplementary Figure 5–8).

## DISCUSSION

Because some acute ischemic stroke patients are unresponsive to first-line endovascular therapy, adjunctive therapies, including emergent angioplasty and antithrombotic pharmaceuticals, are sometimes essential in promoting recanalization. However, the efficacy and safety of antithrombotic pharmaceuticals such as tirofiban remain largely unknown [15].

The randomized, controlled SaTIS Trial demonstrated the safety of tirofiban in treating acute ischemic stroke [26]. For moderate strokes, rates of cerebral hemorrhagic transformation (I/II) and parenchymal hemorrhage (I/II) did not differ between groups treated with or without tirofiban. In addition, low dose tirofiban infusions could improve functional outcomes safely in some moderate acute ischemic stroke patients treated with IV thrombolysis (4 ≤NIHSS ≤18) [27].

Nevertheless, conflicting results have been obtained regarding the safety and efficacy of tirofiban treatment in patients treated with EVT. Kellert et al. found that fatal intracranial hemorrhage increased after tirofiban treatment [14], and Wu YF et al. reported that tirofiban increased sICH risk up to 3-fold when combined with EVT treatment for acute ischemic stroke, especially in the high-dose subgroup [12]. In contrast, tirofiban treatment increased favorable functional outcomes without increasing mortality and sICH both in an observational study [13] and in a recent RCT [16]. Closer examination of mode of administration and dosage might play a crucial role in resolving these conflicting results.

In this meta-analysis, tirofiban treatment had significant benefits in the overall pool of acute ischemic stroke patients treated with EVT, suggesting that the sample sizes examined in previous individual studies may have been too small to detect the efficiency of tirofiban treatment. Our current results suggest that 252 cases in each branch was sufficient and to demonstrate that tirofiban can improve functional outcomes in acute ischemic stroke patients treated with EVT.

Moreover, subgroup analysis showed that standard tirofiban administration (a loading dose followed by maintenance doses) significantly increased favorable functional outcomes compared to a single dose. This difference might be a result of the pharmacokinetics of tirofiban. After the administration of a single dose of tirofiban, plasma concentration measurements indicated a short half-life of 1.4-2.2 hours; that dose failed to prevent thrombogenesis before oral antiplatelets took effect. Furthermore, the indications for tirofiban administration, including multiple retrieval manipulations, residual stenosis, and stent implantation, were independently associated with reocclusion within 24 hours after successful recanalization [28]. This indicates that the risk of reocclusion, and subsequent poorer outcomes, is not reduced by a single dose of tirofiban. Tirofiban therefore may be most effective when administered in an initial loading dose with repeated subsequent maintenance doses to maintain its antiplatelet efficacy.

As part of our analysis, we also grouped patients into 3 subgroups by tirofiban maintenance dosage: 1) low-dose maintenance (loading dose followed by low-dose maintenance); 2) high-dose maintenance (loading dose followed by high-dose maintenance); and 3) unknown maintenance dose. Subgroup analysis indicated that low maintenance doses tirofiban were associated with the greatest improvements in functional outcomes in acute ischemic stroke patients treated with EVT without causing any major adverse events. By comparison, high maintenance doses of tirofiban might potentially increase bleeding events, although that association did not reach statistical significance in subgroup analysis perhaps due to small simple size.

Assessment of the efficacy of tirofiban in recanalizing vessels was complicated by that fact that, in most studies, it was only administered when first-line EVT failed; baseline vessel patency therefore differed systematically between patient groups treated with or without tirofiban. However, a preliminary single-center RCT with small sample size nevertheless found that tirofiban significantly increased recanalization rates and decreased reocclusion rates [16]. Additional randomized control trials should be carried out to characterize the efficacy and safety of different tirofiban doses in combination with EVT for acute ischemic stroke patients.

Several limitations of the current meta-analysis should be considered when interpreting these results. First, although 13 studies were included, many did not provide sufficient data for subgroup analysis; sample sizes in that analysis were therefore small, especially for the high dose maintenance subgroup. Second, most of the included studies were non-randomized, and selection bias was unavoidable because most patients received tirofiban as a rescue therapy after possible failure of recanalization. This selection bias might artificially decrease the efficacy of tirofiban in improving outcomes and decreasing mortality. Third, data regarding systematic bleeding events that were required to assess the safety of tirofiban were not available in all studies. Finally, most of the included studies were carried out in China, and external validity should be established via an international multi-center RCT.

In summary, the present systematic review and meta-analysis revealed that, compared to EVT alone, combined treatment with EVT and tirofiban significantly improved outcomes without increasing mortality or sICH in acute ischemic stroke patients with large vessel occlusion. Furthermore, our results indicate that a loading dose followed by low-dosage maintenance of tirofiban might be the most effective and safest protocol for improving outcomes in acute ischemic stroke patients treated with EVT, although more comprehensive randomized controlled trials are needed to confirm this finding.

## MATERIALS AND METHODS

### Search strategy and selection criteria

The common evidence medicine framework PICO (Patient Population, Intervention, Control, Outcome) was used to examine whether adult acute ischemic stroke patients with a large vessel occlusion who underwent EVT combined with tirofiban had better functional outcomes, higher rates of successful recanalization, and lower rates of mortality and symptomatic intracerebral hemorrhage (sICH) compared to patients who received EVT alone. This meta-analysis was conducted according to PRISMA (Preferred Reporting Items for Systematic Reviews and Meta-Analysis) guidelines [29].

Two neurologists independently and systematically searched PubMed, EMBASE, Web of Science, and The Cochrane Library for studies written in English from January 2000 to October 2019 according to the following strategy and search terms: “((MeSH descriptor: [Stroke] this term only) AND (MeSH descriptor: [tirofiban] this term only))” (Cochrane Library), “(((stroke) AND (tirofiban)) AND ((endovascular therapy) OR (angioplasty) OR (stent)))” (Web of Science), “('brain infarction'/exp OR 'brain infarct' OR 'brain infarction' OR 'cerebral infarct' OR 'cerebral infarction' OR 'cerebrovascular infarction' OR 'cortical infarction' OR 'hemisphere infarct' OR 'hemisphere infarction' OR 'hemispheric infarct' OR 'hemispheric infarction' OR 'infarction, brain' OR 'silent brain infarction' OR 'cerebral artery disease'/exp OR 'anterior cerebral artery infarction' OR 'artery disease, cerebral' OR 'cerebral arterial diseases' OR 'cerebral artery disease' OR 'cerebral artery diseases' OR 'infarction, anterior cerebral artery' OR 'infarction, middle cerebral artery' OR 'infarction, posterior cerebral artery' OR 'intracranial arterial diseases' OR 'middle cerebral artery infarction' OR 'posterior cerebral artery infarction' OR 'occlusive cerebrovascular disease'/exp OR 'brain artery obstruction' OR 'brain artery occlusion' OR 'brain artery thrombosis' OR 'brain phlebothrombosis' OR 'brain thromboembolism' OR 'brain thrombosis' OR 'brain vascular obstruction' OR 'cerebral artery occlusion' OR 'cerebral artery thrombosis' OR 'cerebral thrombosis' OR 'cerebrovascular disease, occlusive' OR 'cerebrovascular obliteration' OR 'cerebrovascular obstruction' OR 'cerebrovascular occlusion' OR 'cerebrovascular occlusion disease' OR 'cerebrovascular occlusive disease' OR 'cerebrovascular thrombosis' OR 'intracranial artery thrombosis' OR 'intracranial thrombosis' OR 'occlusive cerebrovascular disease' OR 'thromboembolism, brain' OR 'thrombosis cerebri' OR 'thrombosis, brain artery' OR 'thrombosis, intracranial' OR 'cardioembolic stroke'/exp OR 'cardioembolic stroke' OR 'carotid artery obstruction'/exp OR 'artery occlusion, carotid' OR 'carotid artery constriction' OR 'carotid artery obstruction' OR 'carotid artery occlusion' OR 'carotid artery occlusive disease' OR 'carotid artery stenosis' OR 'carotid obliteration' OR 'carotid occlusion' OR 'carotid stenosis') AND ('tirofiban'/exp OR 'aggrastat' OR 'aggrastet' OR 'agrastat' OR 'l 700462' OR 'mk 0383' OR 'mk 383' OR 'n (butylsulfonyl) o [4 (4 piperidinyl) butyl] tyrosine' OR 'tirofiban' OR 'tirofiban hydrochloride' OR 'tirofiban hydrochloride monohydrate') AND ('percutaneous thrombectomy'/exp OR 'catheter based thrombectomy' OR 'catheter directed thrombectomy' OR 'endovascular embolectomy' OR 'endovascular thrombectomy' OR 'percutaneous thrombectomy' OR 'percutaneous transluminal angioplasty'/exp OR 'angioplasty, balloon' OR 'angioplasty, percutaneous transluminal' OR 'angioplasty, transluminal' OR 'balloon angioplasty' OR 'dotter artery dilatation' OR 'percutaneous angioplasty' OR 'percutaneous transluminal angioplasty' OR 'percutaneous transluminal artery dilatation' OR 'transluminal angioplasty' OR 'transluminal artery dilatation' OR 'stent'/exp OR 'stent' OR 'stenting' OR 'stents')” (EMBASE), “(((((“cerebral infarction ” OR "brain infarction" OR "acute ischemic stroke" OR "acute ischaemic stroke" OR "stroke" OR "anterior circulation infarction" OR "anterior circulation cerebral infarction" OR "posterior circulation cerebral infarction" OR "posterior circulation infarction" OR "carotid artery occlusion" OR "middle cerebral artery occlusion" OR "anterior cerebral artery occlusion" OR "posterior cerebral artery occlusion" OR "vertebrobasilar artery occlusion" OR "basilar artery occlusion" OR "cerebral artery embolism" OR "intracranial atherosclerosis stenosis"))) AND (((“Thrombectomy” OR “Thrombectomies” OR “Percutaneous Aspiration Thrombectomy” OR “Aspiration Thrombectomies, Percutaneous” OR “Aspiration Thrombectomy, Percutaneous” OR “Percutaneous Aspiration Thrombectomies” OR “Thrombectomies, Percutaneous Aspiration” OR “Thrombectomy, Percutaneous Aspiration” OR “Aspiration Thrombectomy” OR “Aspiration Thrombectomies” OR “Thrombectomies, Aspiration” OR “Thrombectomy, Aspiration” OR “angioplasty” OR “stenting” OR "endovascular therapy" OR "endovascular treatment")))) AND (“N-(Butylsulfonyl)-O-[4-(4-piperidynyl)butyl]-L-tyrosine” OR “tirofiban” OR “glycoprotein IIbIIIa antagonist” OR “glycoprotein IIb/IIIa antagonist” OR “Aggrastat” OR “L 700462” OR “MK-383”))” (PubMed).

References generated from these searches were imported into the reference manager EndNote X9.3.1 (Thompson Reuters, Philadelphia, PA) and duplicate references were removed. Journal article titles and abstracts were then systematically screened for studies comparing outcomes of interest between EVT + tirofiban and EVT-only patients by 2 neurologists independently according to the following inclusion criteria: (1) randomized controlled trials and observational studies (case-control studies and cohort studies); (2) evaluation of the efficacy and safety of tirofiban on acute ischemic stroke patients treated with EVT. Studies were excluded if they (1) were unpublished studies or conference abstracts; (2) contained duplicate data on patients reported in other studies; (3) lacked outcome data beyond hospitalization; (4) did not report data on both EVT + tirofiban and EVT-only patient groups; (5) were case-series with <10 patients.

### Data extraction and validity assessment

Two reviewers independently extracted data; discrepancies were resolved by consulting the third senior neurointerventionist. In case of incomplete or unclear data, authors were contacted where possible. The name of first author, published year, country, design, sample size, average age, sex ratio, time from symptom onset to groin puncture/recanalization, intravenous thrombolysis, first-line EVT method, and tirofiban administration strategy were extracted using prespecified forms. The primary endpoint of the analysis was 90-day favorable functional outcome defined as mRS ≤2. The secondary efficacy outcome was recanalization rate defined by modified Thrombolysis in Cerebral Infarction (mTICI) 2b or 3. Safety outcomes included the composite of death and symptomatic intracranial hemorrhage (sICH). Studies were stratified pre-hoc based on tirofiban administration strategy. Study validity and risk of bias were evaluated using The Newcastle-Ottawa Scale (NOS) for observational studies and The Cochrane Collaboration methods for RCTs.

### Subgroup analysis based on tirofiban dosage and mode of administration

Given the short half-life of tirofiban, patients were divided into 1) single dose and 2) loading + maintenance dose subgroups depending on whether maintenance tirofiban doses were administered following the initial dose. Single dose was defined as patients who received a single dose via catheter or intravenous injection without subsequent tirofiban maintenance doses. The loading + maintenance group was further divided into 2 subgroups: 1) Low-dose, defined as a maintenance dosage no greater than 0.1 μg/kg/min, and 2) High-dose, defined as a maintenance dosage greater than 0.1 μg/kg/min.

### Data analysis and synthesis

Odds ratios (OR) were calculated for individual studies and pooled according to Mantel-Haenszel random-effect methods (with 95% confidence intervals) using Review Manager 5.3.5 (The Cochrane Collaboration, Denmark). Statistical significance was defined by *p* ≤ 0.05. A meta-analysis was conducted for each outcome of interest. Significant heterogeneity was defined by both a χ^2^ value with a *p* value < 0.10 and an I^2^ value greater than 50%. Bias due to small study sizes and/or publication bias (i.e. whether studies reporting significant effects are more likely to be published than those reporting no effects) were assessed via visual inspection of funnel plots and a Peters test using Stata 15.0 (Statacorp, USA).

### Availability of data and material

The datasets used and analyzed in the current study are available from the corresponding author upon reasonable request.

## Supplementary Material

Supplementary Figures

Supplementary Table 1
