# Supplementary material for: Sequential tirofiban infusions combined with endovascular treatment may improve outcomes in acute ischemic stroke - a meta-analysis
Source: Aging (Albany NY). 2021 Feb 11;13(4):5426–41. doi: 10.18632/aging.202473 (PMC7950282; doi:10.18632/aging.202473)
Supplement: Supplementary Figures [file aging-13-202473-s001.pdf]

SUPPLEMENTARY FIGURES

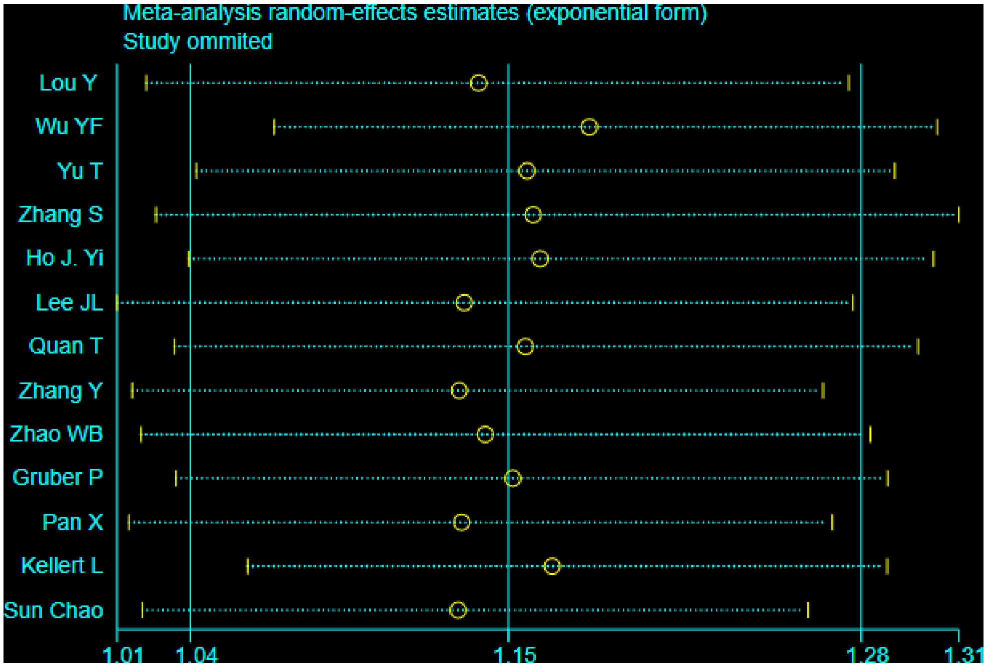

Supplementary Figure 1. The results of a sensitivity analysis for 90-day favorable functional outcomes in acute ischemic stroke patients who underwent endovascular therapy.

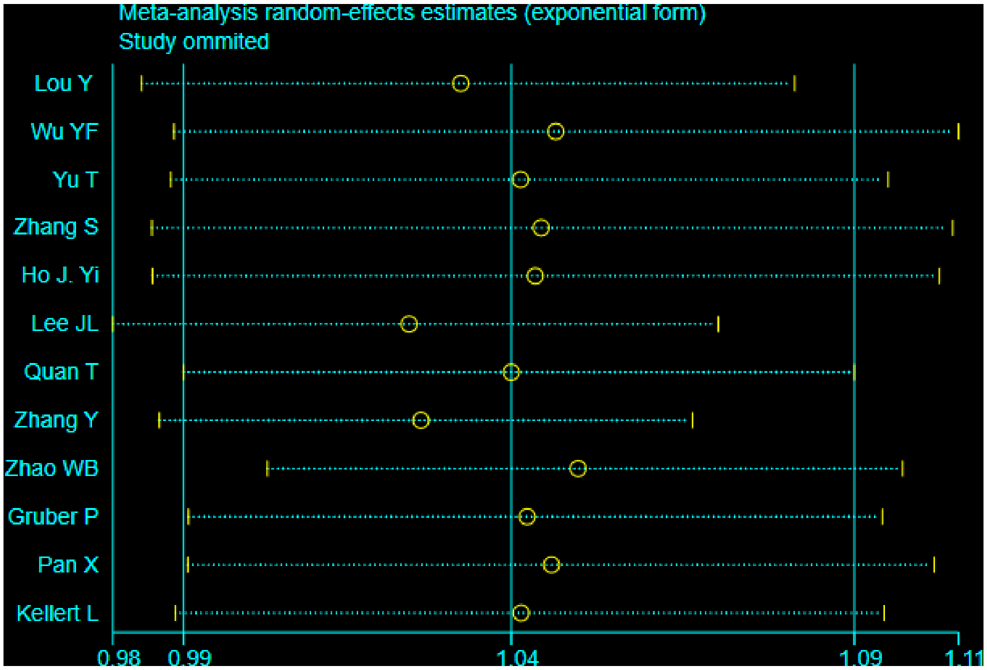

Supplementary Figure 2. The results of a sensitivity analysis for recanalization rates in acute ischemic stroke patients who underwent endovascular therapy.

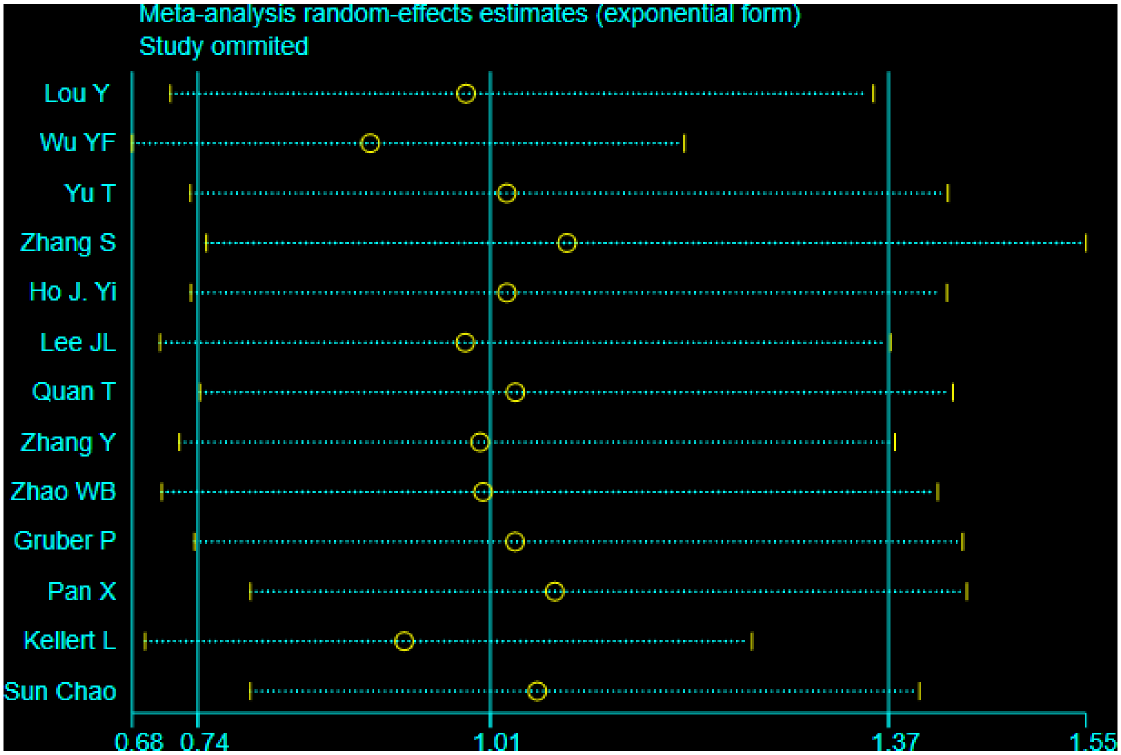

Supplementary Figure 3. The results of a sensitivity analysis for sICH rates in acute ischemic stroke patients who underwent endovascular therapy.

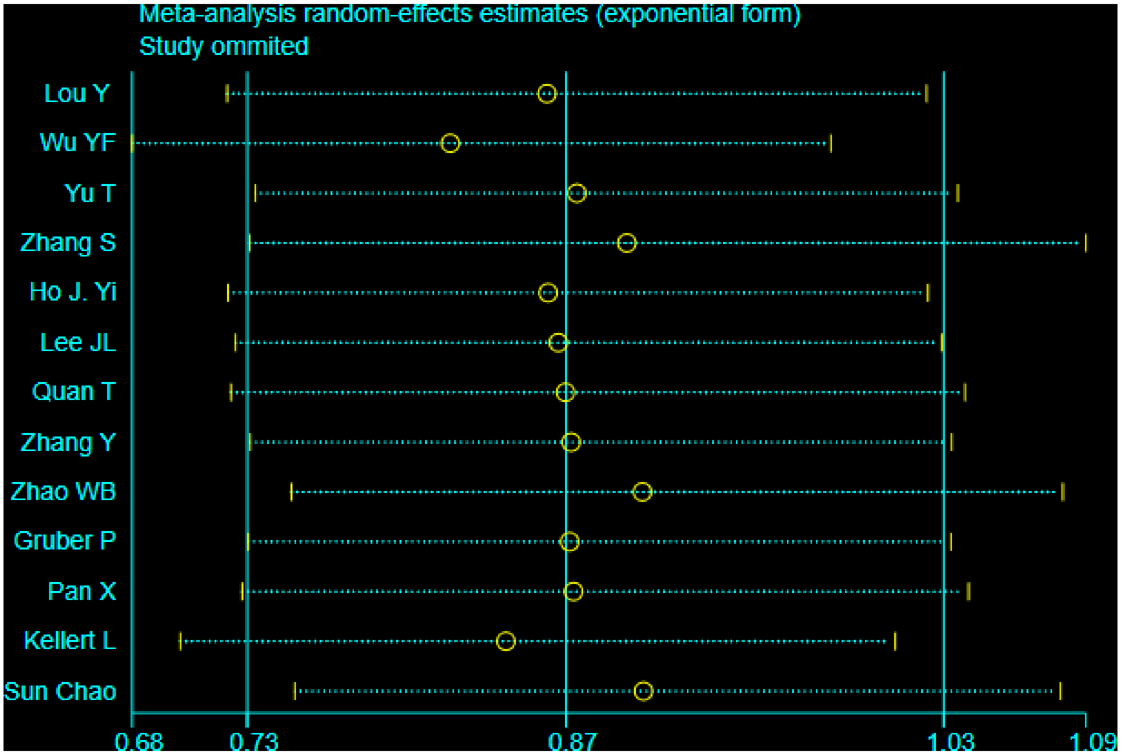

Supplementary Figure 4. The results of a sensitivity analysis for 90-day mortality in acute ischemic stroke patients who underwent endovascular therapy.

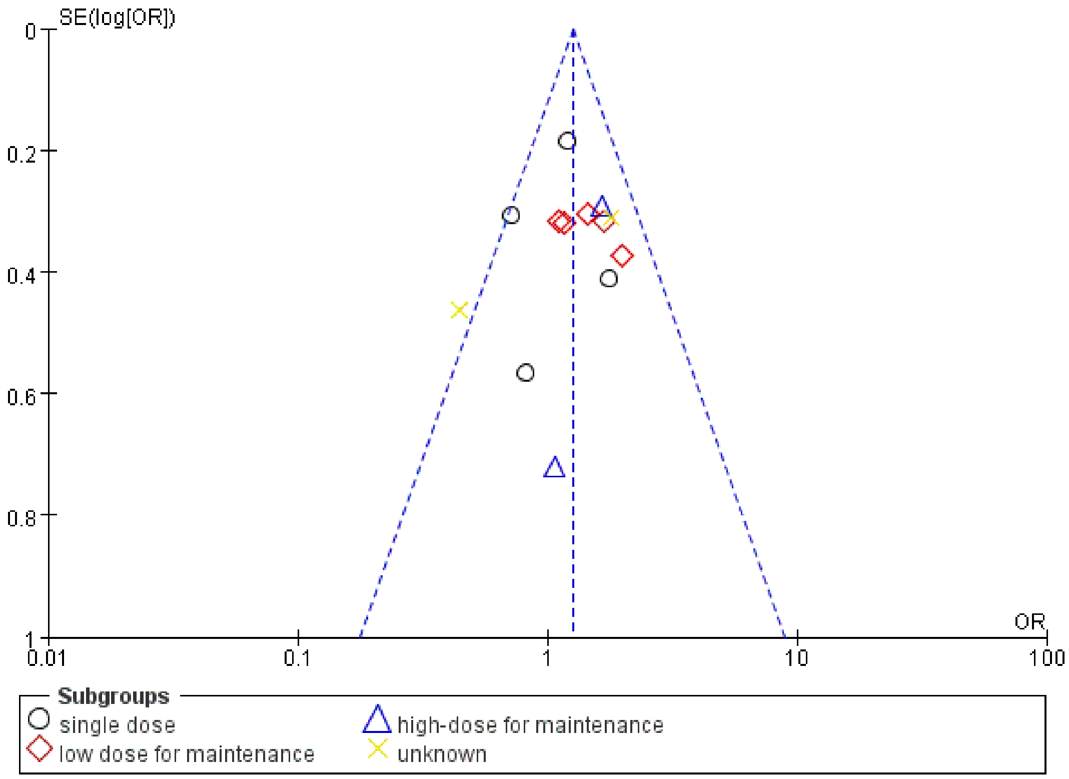

Supplementary Figure 5. A funnel plot for assessing publication bias in regards to studies reporting 90-day favorable functional outcomes in acute ischemic stroke patients who underwent endovascular therapy.

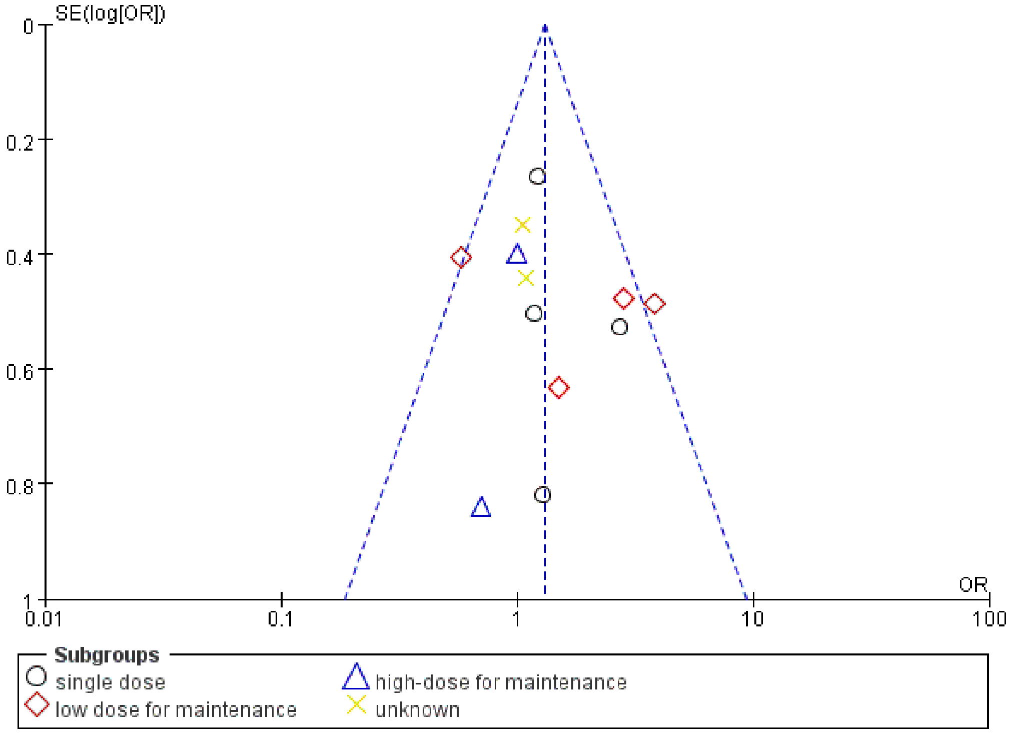

Supplementary Figure 6. A funnel plot for assessing publication bias in regards to studies reporting recanalization rates in acute ischemic stroke patients who underwent endovascular therapy.

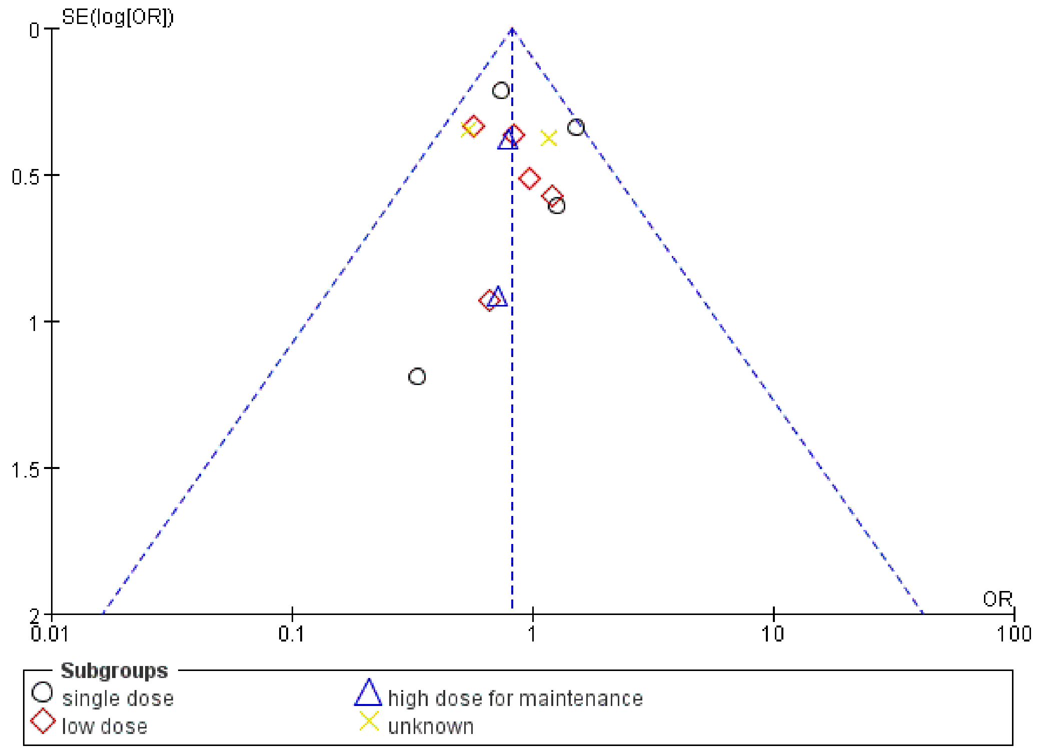

Supplementary Figure 7. A funnel plot for assessing publication bias in regards to studies reporting 90-day mortality in acute ischemic stroke patients who underwent endovascular therapy.

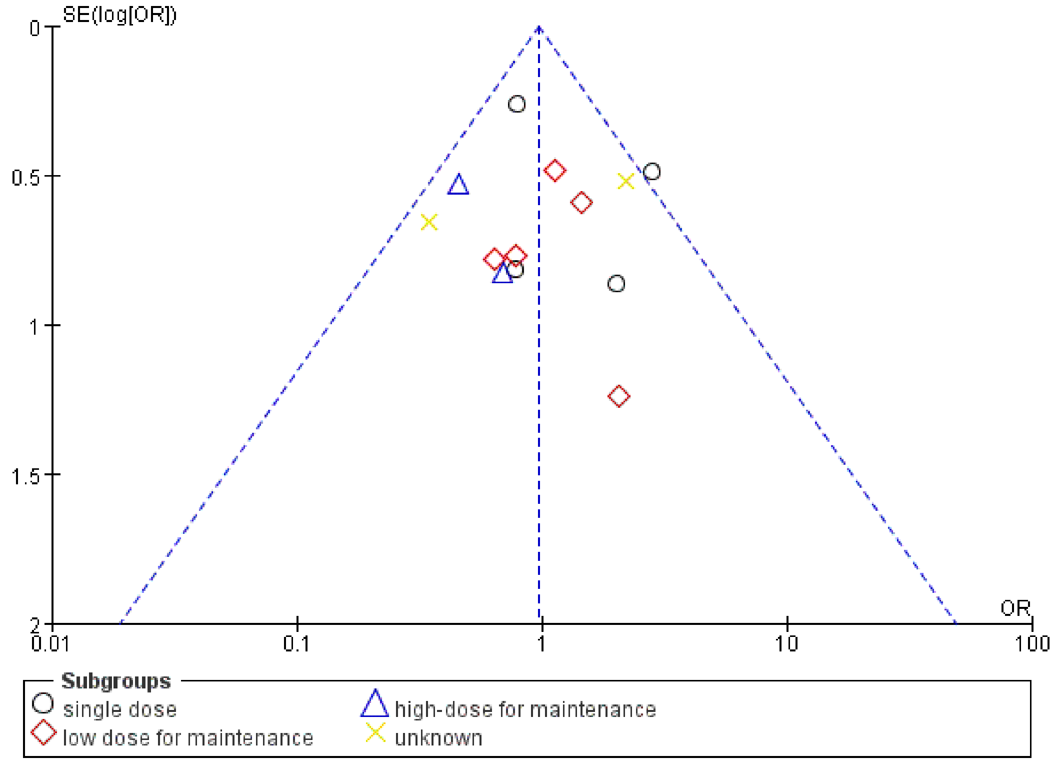

Supplementary Figure 8. A funnel plot for assessing publication bias in regards to studies reporting sICH in acute ischemic stroke patients who underwent endovascular therapy.
