# Supplementary material for: Sequential tirofiban infusions combined with endovascular treatment may improve outcomes in acute ischemic stroke - a meta-analysis
Source: Aging (Albany NY). 2021 Feb 11;13(4):5426–41. doi: 10.18632/aging.202473 (PMC7950282; doi:10.18632/aging.202473)
Supplement: Supplementary Table 1 [file aging-13-202473-s002.docx]

**Supplementary Table 1. Main characteristics of studies included in the meta-analysis.**

| **Study** | **Year** | **Region** | **Study type** | **Lesion site** | | **First-line EVT** | **Tirofiban administration strategy** | **Assigned dose subgroup** | **Sample size** | | **Mean patient age** | | **Male%** | | **NIHSS onset** | | **Time from onset to groin puncture** | | **Time from onset to recanalization** | | **% of patients with IV thrombolysis** | |
| --- | --- | --- | --- | --- | --- | --- | --- | --- | --- | --- | --- | --- | --- | --- | --- | --- | --- | --- | --- | --- | --- | --- |
|  |  |  |  | Anterior | Posterior |  |  |  | Tirofiban | Control | Tirofiban | Control | Tirofiban | Control | Tirofiban | Control | Tirofiban | Control | Tirofiban | Control | Tirofiban | Control |
| Sun C | 2019 | China | cohort | 146 | 49 | MT | 0.25-0.5mg IA loading dose,  undisclosed maintenance dose | NA  (Not Addressed) | 71 | 124 | 66.2 | 66.3 | 71.80% | 69.40% | 14  (9–20) | 15.5  (11–20) | 340 (215–505)  (min) | 301 (218–433)  (min) | 440 (290–602)  (min) | 375 (305–540)  (min) | 33.80% | 44.40% |
| Yi HJ | 2019 | Korea | cohort | 303 | 24 | MT | 0.25-1mg IA loading dose,  0.05ug/kg/min maintenance dose | low dose  maintenance | 47 | 280 | 73.4 | 69.1 | 59.60% | 56.10% | 10.5  (1-18) | 9.6  (1-18) | NA | NA | NA | NA | 46.80% | 39.60% |
| Zhang Y | 2019 | China | RCT | 67 | 53 | MT | 0.2 ug/kg/min *15min loading dose,  0.1ug/kg/min maintenance | low dose  maintenance | 60 | 60 | 56.4 | 56.4 | 70.00% | 70.00% | 14.3±6.4 | 14.3±6.4 | 4.6±3.1(h) | 4.6±3.1(h) | NA | NA | 28.33% | 28.33% |
| Zhang S | 2019 | China | cohort | 632 | 0 | MT | 0.25-1mg IA single dose | single dose | 154 | 478 | 64.3 | 64.6 | 59.70% | 57.70% | 15  (12.0–20.0) | 17  (13.0–21.0) | NA | NA | 397 (299–499)  (min) | 381 (307–470)  (min) | 29.22% | 32.64% |
| Quan T | 2019 | China | case-control | 0 | 159 | MT/ Aspiration | 0.25-0.5 mg IA loading dose，  0.2–0.25 mg/h ivp maintenance | low dose  maintenance | 85 | 74 | NA | NA | NA | NA | NA | NA | NA | NA | NA | NA | NA | NA |
| Pan X | 2019 | China | cohort | 75 | 36 | MT | 0.15μg/kg/min maintenance | high dose  maintenance | 82 | 129 | 69.5 | 74.0 | 63.40% | 61.20% | 15.0  (10.0-19.0) | 14.0  (10.5-20.0) | NA | NA | NA | NA | 34.15%* | 72.87% |
| Lou Y | 2019 | China | cohort | 91 | 8 | MT | 50ug/min (50 ug/mL),  total amount of 50 ug/kg, pre-EVT single dose | single dose | 56 | 43 | 64.0 | 68.0 | 64.30% | 55.80% | 16.41±6.22 | 15.72±5.28 | NA | NA | NA | NA | NA | NA |
| Gruber P | 2019 | Switzerland | cohort | 32 | 0 | Stenting | 10ug/kg IA loading dose，  0.15ug/kg/min ivp maintenance | high dose  maintenance | 18 | 14 | 66.0 | 68.0 | 72.00% | 71.00% | 14  (9–19) | 18  (8–20) | 237 (187–285)  (min) | 243 (187–295)  (min) | NA | NA | NA | NA |
| Yu T | 2018 | China | cohort | 47 | 7 | MT | <0.5mg IA single dose | single dose | 26 | 28 | 70.3 | 67.8 | 46.20% | 53.60% | 13.42±4.28 | 12.57±4.95 | 202.31±100.96  (min) | 199.00±90.68  (min) | 300.77±100.12  (min) | 304.39±99.48  (min) | 30.77% | 28.57% |
| Wu YF | 2018 | China | cohort | 176 | 48 | MT | strategized single IA dose:  3ug/kg, 6.7ug/kg, 10ug/kg | single dose | 94 | 124 | 70.0 | 71.5 | 38.30% | 45.20% | 18  (12-22) | 18  (13-23) | 314 (240-360)  (min) | 295 (220-360)  (min) | NA | NA | 15.96%* | 30.08% |
| Zhao WB | 2017 | China | cohort | 118 | 62 | MT | 0.25-0.5mg IA loading dose，  0.2-0.25mg/h ivp maintenance | low dose  maintenance | 90 | 90 | 61.8 | 60.8 | 77.00% | 67.00% | 21  (14–32) | 19  (15–26) | 314 (235–379)  (min) | 300 (236–375)  (min) | 388 (321–421)  (min) | 372 (311–436)  (min) | 24.44% | 22.22% |
| Lee JL | 2017 | Germany | case-control | 195 | 0 | Stenting | 1.25mg IV loading dose，  0.1ug/kg/min ivp maintenance | low dose  maintenance | 60 | 135 | 71.0 | 76.0 | 63.33% | 40.74% | 11  (6–16) | 15  (11–18) * | 175 (139–238)  (min) | 183 (143–294)  (min) | 263 (217–332)  (min) | 263 (217–332)  (min) | 80.00% | 85.93% |
| Kellert L | 2013 | Germany | cohort | 128 | 34 | MT | NA | NA | 50 | 112 | 64.5 | 67.3 | NA | NA | 18  (13, 35) | 19  (16, 23) | 140 (93, 270)  (min) | 150 (93, 280)  (min) | 254 (157, 353)  (min) | 239 (164, 386)  (min) | NA | NA |

* *p*<0.01. NA- Not Addressed. EVT- Endovascular therapy. MT- Mechanical Thrombectomy. IA- Intra-artery. IV- Intra-venous. Ivp- Intravenous pump.
